# Radiotherapy in Cutaneous Anaplastic Large Cell Lymphoma

**DOI:** 10.4103/0974-2077.63258

**Published:** 2010

**Authors:** Raj Hegde

**Affiliations:** *William Buckland Radiotherapy Centre, Alfred Hospital, Melbourne 3004, Victoria, Australia. E-mail: krheg@hotmail.com*

Sir,

I was delighted to see the article “Primary cutaneous anaplastic large cell lymphoma (CALCL) arising from lymphomatoid papulosis, responding to low dose methotrexate,” published in the *Journal of Cutaneous and Aesthetic Surgery*, 24 December 2009 issue. Usually, this type of cutaneous lymphomas have an excellent prognosis with more than 90% cure rate with local therapy alone.[1] In nearly 25% of cases, spontaneous regression of the primary cutaneous form occurs despite the high-grade anaplastic cytology of neoplastic lymphocytes.

Systemic treatment is usually recommended for multifocal lesions. The guidelines of the British Association of Dermatologists, UK, do not recommend chemotherapy in patients with early stage IA, IB, or IIA disease.[2] The treatment of a localized CALCL usually involves localized radio therapy or surgical excision.[3]

The cutaneous T-cell lymphoma variants are very radiosensitive malignancies. Individual thick plaques, eroded plaques, or tumors can be treated successfully with low-dose superficial orthovoltage radio therapy or electron beams.[3] The range of radiation dose delivered is 34–44 Gy, in 2- to 3-Gy fractions, five fractions per week. Large tumors may be treated by electron beams, the choice of energy being dependent on the tumor size and thickness. Radiotherapy is often used with other therapeutic modalities such as PUVA. Closely adjacent and overlapping fields can often be retreated because of the low doses used.
